# Topographical Quantification of Hyper-Reflective Foci May Predict the Development of Macular Atrophy in Patients With Neovascular Age-Related Macular Degeneration

**DOI:** 10.1167/iovs.65.14.45

**Published:** 2024-12-31

**Authors:** Pasquale Viggiano, Giacomo Boscia, Giuseppe Giannaccare, Michele Santoro, Giovanni Petrara, Ciro Borriello, Enrico Borrelli, Michele Reibaldi, Maria Oliva Grassi, Giovanni Alessio, Francesco Boscia

**Affiliations:** 1 Department of Translational Biomedicine Neuroscience, University of Bari “Aldo Moro,” Bari, Italy; 2 Eye Clinic, Department of Surgical Sciences, University of Cagliari, Cagliari, Italy; 3 Department of Surgical Sciences, University of Turin, Turin, Italy

**Keywords:** neovascular age-related macular degeneration (nAMD), optical coherence tomographic (OCT), macular neovascularization (MNV), hyper-reflective foci (HRF)

## Abstract

**Purpose:**

The purpose of this study was o examine the optical coherence tomographic (OCT) characteristics of hyper-reflective foci (HRF) in patients with neovascular age-related macular degeneration (nAMD) and to assess the potential of HRF as a predictive factor for the development of macular atrophy following anti-vascular endothelial growth factor (anti-VEGF) therapy.

**Methods:**

This was a retrospective analysis of 61 treatment-naïve eyes diagnosed with exudative AMD and type 1 macular neovascularization (MNV). The HRF was identified in the inner retina and outer retina layers, and the treatment response of HRF was documented. An analysis was conducted to explore the association between HRF and the development of macular atrophy.

**Results:**

The number of HRF in the inner retina and outer retina layers showed significant reduction after 12 months of anti-VEGF treatment (*P* = 0.002 and *P* < 0.0001, respectively). Similarly, compared with baseline, the number of HRFs in the inner retina and outer retina layers was significantly reduced after 24 months of anti-VEGF treatment (*P* = 0.002 and *P* < 0.0001, respectively). Moreover, the multivariate linear regression analysis revealed that the most substantial associations observed with the development of macular atrophy after 12 months were specifically tied to the number of HRFs in the outer retina (*P* = 0.039) at the baseline visit. This finding was confirmed after 24 months of anti-VEGF treatment (*P* = 0.007).

**Conclusions:**

After only 1 year of antiangiogenic therapy, there was a significant decrease in HRFs observed across all retinal layers. This reduction persisted even after 2 years of anti-VEGF treatment. Notably, the quantity of HRFs in the outer retina at baseline exhibited a correlation with the development of macular atrophy that endured at both the 1-year and 2-year follow-ups after anti-VEGF treatment.

Neovascular age-related macular degeneration (nAMD) stands as the predominant cause of blindness in developed countries.^1^ Whereas therapy with anti-vascular endothelial growth factor (anti-VEGF) has demonstrated effectiveness in treating nAMD,^2^^,^^3^ it is important to note that the treatment response can vary significantly among individual patients. There has been a significant exploration for biomarkers that could potentially serve as predictive indicators for treatment response or ultimate outcomes in order to customize a treatment plan according to a prognosis.^4^ For instance, reticular pseudodrusen have been identified as a significant structural biomarker with high prognostic value for the development of geographic atrophy in AMD.^5^^,^^6^

The advent of structural optical coherence tomography (OCT) has facilitated the acquisition of quantitative measurements pertaining to the neuroretina and alterations associated with AMD. These include assessments of intraretinal fluid (IRF), subretinal fluid (SRF), subretinal hyper-reflective material (SHRM), pigment epithelial detachment (PED), and the integrity of the retinal pigment epithelium (RPE), along with the evaluation of macular neovascularization (MNV).^7^^–^^9^

Using structural OCT, it is possible to identify intraretinal and choroidal hyper-reflective foci (HRF) in different disorders, including diabetic retinopathy, retinal vein occlusion, and AMD.^10^^–^^13^ Intraretinal HRF are distinct, well-defined dots with reflectivity equal to or greater than that of the RPE within the neurosensory retina.^14^ Various hypotheses have been proposed regarding the HRF origin, including activation of microglia in an inflammatory environment,^15^ extravasation of lipoproteins,^10^ and migration of RPE cells.^16^

In AMD research, there has been a thorough examination of intraretinal HRF due to their correlation with the advancement and worsening of late AMD, particularly in cases of geographic atrophy. However, earlier investigations have also assessed the potential of HRF as a biomarker for visual outcomes in patients with nAMD with conflicting results. Lee et al.^17^ indicated that HRF in the subretinal layer might offer improved predictability for the final visual acuity. In contrast, Framme et al.^18^ observed the regression of HRF after anti-VEGF treatment, highlighting an inverse correlation between the presence of HRF at baseline and eventual visual acuity in patients with nAMD, especially in cases of polypoidal choroidal vasculopathy (PCV). Moreover, Nassisi et al.^19^ observed that HRF within drusenoid lesions (DLs) served as a risk factor for the development of atrophy in the fellow eyes of patients newly diagnosed with MNV.

These conflicting results may be attributed to the fact that HRF in different retinal layers could have distinct origins and clinical presentations, leading to varying predictability concerning anatomic or functional outcomes. Additionally, considering previous findings that HRF situated in the outer retinal layer were associated with a less favorable visual prognosis and accompanied by external limiting membrane (ELM) and photoreceptor disruption in conditions such as diabetic macular edema and retinal vein occlusion,^20^^,^^21^ we postulated that examining HRF across distinct retinal layers would be valuable in identifying the most impactful prognostic factors.

Therefore, this study aimed to assess the response of HRF in both inner and outer retinal layers following the administration of anti-VEGF therapy in eyes affected by nAMD. More importantly, we explored whether the amount and location of HRF may be biomarkers for the development of macular atrophy over a 2-year period. Because macular atrophy is a frequent and sight-threatening complication of neovascular AMD,^22^^,^^23^ it would be important to investigate factors associated with its development.

## Methods

### Study Participants

This retrospective cohort study was carried out within the retina service of the Department of Translational Biomedicine Neuroscience at the University of Bari “Aldo Moro.” Medical records collected from January 2017 to January 2022 at the Medical Retina and Imaging Unit of the Department of Translational Biomedicine Neuroscience (University of Bari “Aldo Moro”) were reviewed. Ethical considerations adhered to the principles outlined in the Declaration of Helsinki, and the study protocol received approval from the institutional review board of the Department of Translational Biomedicine Neuroscience at the University of Bari “Aldo Moro.”

In this study, patients with treatment-naïve exudative nAMD with type 1 MNV were identified. The diagnosis of nAMD and type 1 MNV was established based on the observations from OCT, OCT angiography (OCTA) and fluorescein angiography.^24^^,^^25^

The exclusion criteria at baseline included: (1) evidence of aneurysmatic type 1, type 2, or type 3 MNV; (2) any other underlying disease associated with type 1 MNV; (3) evidence of macular complications including RPE tear, macular atrophy or fibrosis, as determined by OCT findings and clinical examination^26^; and (4) any optic neuropathy including glaucoma. To be included, patients were also required to have at least 1 visit every 3 months covering a period of 2 years following the initiation of the anti-VEGF treatment. All the analyzed patients were treated with either 0.5 mg ranibizumab or 2 mg aflibercept according to the decision of the physician. The treatment started with three monthly injections as the loading phase, followed by subsequent injections administered on an as-needed basis, determined by clinical findings recorded during each follow-up visit.

Exclusion criteria at follow-up visits included: (i) absence of OCT scans obtained with the follow-up/reference scan function; (ii) occurrence of macular complication including subretinal fibrosis or RPE tear; (iii) presence of poor-quality images in which it was not allowed to evaluate the occurrence of macular atrophy or quantify HRF; and (iv) a number of anti-VEGF injections lower than six in the first year and lower than four in the second year.

The population that fulfilled the inclusion and exclusion criteria at both baseline and follow-up visits comprised 61 eyes from 61 patients. This cohort study underwent treatment with intravitreal injections of ranibizumab or aflibercept for AMD-associated type 1 MNV.

### Ophthalmology Assessment

All the included subjects underwent a complete ophthalmologic examination at both the baseline and follow-up visits, including measurement of best-corrected visual acuity (BCVA) and intraocular pressure, and dilated ophthalmoscopy. BCVA was measured using standardized Early Treatment Diabetic Retinopathy Study (ETDRS) charts at a distance of 4 meters. Intraocular pressure was measured using Goldmann applanation tonometry. Additionally, all patients underwent structural OCT imaging with the Spectralis OCT (SPECTRALIS; Heidelberg Engineering, Inc., Heidelberg, Germany) device. The following visits were included in the analysis: (i) the baseline visit that was conducted the day of the first anti-VEGF injection; (ii) the “12-month follow-up visit” which was performed 12 ± 2 months after the first injection of anti-VEGF; and (iii) the “24-month follow-up visit” carried out at 24 ± 2 months after the first anti-VEGF injection. BCVA was transformed into the logarithm of the minimal angle of resolution (logMAR) equivalents for statistical analyses.

### Imaging Grading

All OCT scans at both baseline and follow-up visits were acquired using a Spectralis OCT (SPECTRALIS; Heidelberg Engineering, Inc., Heidelberg, Germany) device using a volume scanning protocol of 49 B-scans covering an area of 6 × 6 mm.

**Figure 1.**
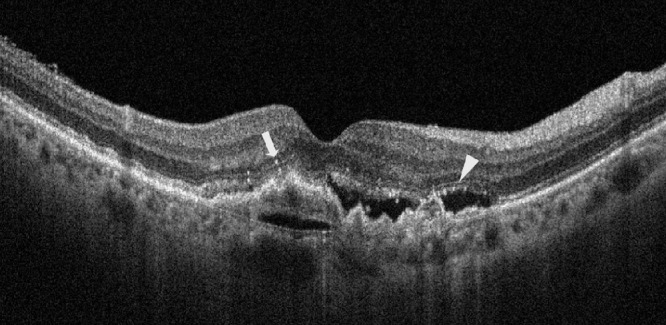
Representative case of AMD-associated treatment-naïve type 1 MNV with HRF. A 69-year-old female patient presented with nAMD. OCT image showed SRF and PED. HRFs were observed in both the inner retina (*arrow*) and outer retina (*arrowhead*) layers.

The analysis at baseline and follow-up visits (12-month and 24-month visits) included the examination of the following OCT findings:

• Central foveal thickness (CFT), which was automatically calculated as the average retinal thickness within a circle with a 500 µm radius, centered on the fovea. This calculation was based on the volume scan data encompassing the specified circle area.
• Presence of IRF, which was considered to be present when there was an accumulation of fluid leading to retinal thickening and the formation of cystoid spaces on structural OCT.
• Presence of SRF was considered present when there was an accumulation of fluid under the retina, leading to the separation of the neuroretina from the RPE on structural OCT.
• Presence of SHRM was identified as hyper-reflective material within the subretinal space.
• Presence and number of HRFs that were characterized as well-circumscribed, discrete lesions with reflectivity equal to or greater than that of the RPE band. HRFs were defined with a diameter ranging from 20 to 40 µm. Hyper-reflective dots smaller than 20 µm were considered as noise signals and were not included in the analysis. Additionally, large confluent hyper-reflective focus clumps, corresponding to hard exudates on the fundus, were excluded from the study.^17^ HRFs were divided based on the topographical location yielding two groups of HRF, as follows: “HRF in the inner retina” (from the outer nuclear layer to the internal limiting membrane), and “HRF in the outer retina” (from above the RPE to the external limiting membrane; Fig. 1).
• The presence of IRF, SRF, SHRM, and macular atrophy, were independently evaluated by two experienced graders (authors P.V. and E.B.). In case of disagreement, a third senior grader made the final decision (author F.B.).
• A retinal specialist (author P.V.) manually counted the HRFs within the area of 1500 µm centered on the fovea.^27^ To gauge the reproducibility of our measurements, a second retinal specialist (author E.B.) manually counted the HRFs in 15 eyes. The intraclass correlation coefficient was calculated to assess the interobserver concordance of HRF counting.

**Figure 2.**
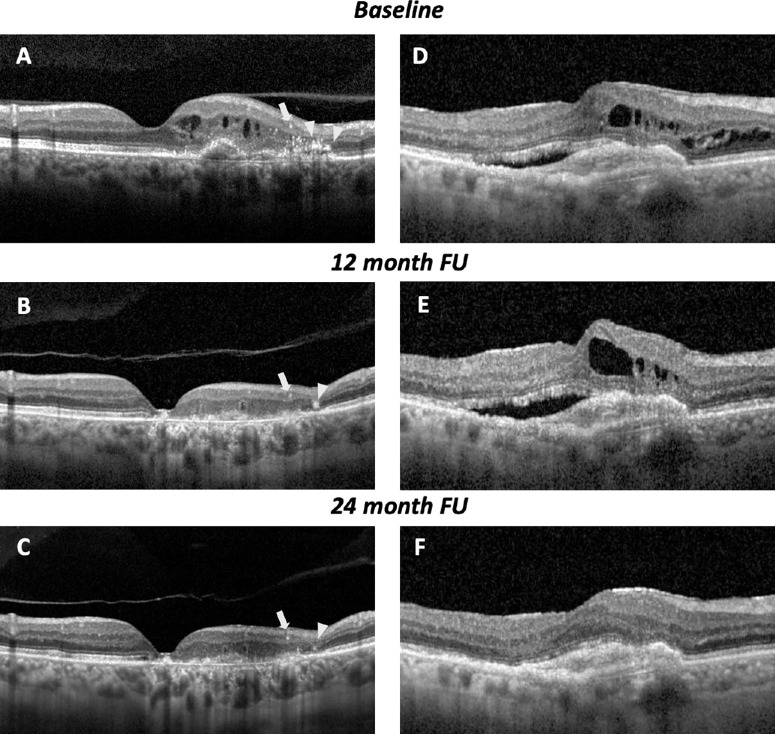
Representative case of AMD-associated treatment-naïve type 1 MNV with HRFs at baseline and follow-up visits. (**A**) A 71-year-old male patient presented with nAMD. OCT image at baseline showed intraretinal fluid, SHRM, and PED. Central foveal thickness was 315 µm. HRFs were observed in the inner (*arrow*) and outer retina levels (*arrowhead*). (**B**) After 12 months of intravitreal injection of ranibizumab (8 injections), OCT showed IRF resolved, the height of PED decreased, and there was a presence of cRORA. Central foveal thickness was 222 µm. (**C**) After 24 months of intravitreal injection of ranibizumab (11 injections), OCT demonstrated an enlarged area of atrophy accompanied by the presence of outer retinal tubulations. Representative case of AMD-associated treatment-naïve type 1 MNV without HRFs at baseline and follow-up visits. (**D**) A 74-year-old male patient presented with nAMD. OCT image at baseline revealed SRF, IRF, and PED. Central foveal thickness was 377 µm. (**E**) After 12 months of intravitreal injection of aflibercept (7 injections), OCT showed persistent SRF, IRF, and PED without the presence of cRORA. Central foveal thickness was 389 µm. (**F**) After 24 months of intravitreal injection of aflibercept (10 injections), OCT demonstrated resolution of IRF and SRF, with a decrease in the height of PED.

At the follow-up visits, the presence of macular atrophy was also graded (^1^Fig. 2). This was defined as an increased hypertransmission of OCT signal into the choroid with a complete RPE and outer retinal atrophy (cRORA) defined using the Classification of Atrophy Meeting (at least 250 mm in diameter) with an overlying RPE defect and thinning of the outer retina.^22^^,^^28^ Clinical data were gathered at baseline and during each subsequent follow-up visit after the initial injection.

### Statistical Analysis

Statistical computations were carried out using the Statistical Package for Social Sciences (version 23.0; SPSS Inc., Chicago, IL, USA). Descriptive statistics for parametric numeric data involved calculating the mean and standard deviation, whereas categorical variables were expressed as percentages. To identify deviations from a normal distribution, a Shapiro-Wilk’s test was executed for all variables. To conduct pairwise comparisons of BCVA, topographical HRF, and CFT at each time point, a paired *t*-test was used, and Bonferroni post hoc corrections were applied. Pearson correlation was utilized to examine the correlation in the topographical distribution of HRFs during each time visit. To investigate the predictive capability of various variables and their association with the development of macular atrophy at each time point, we used a regression analysis while adjusting for age and sex. Initially, univariate regression analysis was conducted, and parameters with a *P* value of < 0.1 from this analysis were subsequently included in the multiple logistic regression analysis to identify independent and significant factors. Biomarkers, including IRF, SRF, topographical HRF amount, and CFT, were incorporated for analysis. A significance level of *P* < 0.05 was considered statistically significant. The unweighted Kappa (k) statistic test was performed to evaluate the agreement between graders concerning qualitative features in OCT. In addition to the main analyses, we conducted a subgroup analysis to evaluate potential differences between treatment types. Patients were separated into two groups based on their anti-VEGF treatment (ranibizumab, *n* = 19 and aflibercept, *n* = 42). The primary statistical analyses were repeated for each subgroup to assess the consistency of our findings across different anti-VEGF agents.

## Results

### Characteristics of Patients Included in the Analysis

Sixty-one eyes of 61 patients scheduled for anti-VEGF therapy for AMD-associated treatment-naïve type 1 MNV (mean age = 78.7 ± 9.7 years) were included. Table 1 provides an overview of the demographic and clinical characteristics of this study cohort.

**Table 1.** Baseline Characteristics of Patients With nAMD Type 1 MNV

Number of patients, *n*	61
Number of eyes, *n*	61
Age, y	78.7 ± 9.7
Gender, M⁄F	24/37
Initial BCVA, logMAR	0.5 ± 0.6
Initial CFT, µm	333.1 ± 109.8
Intraretinal fluid at baseline, *n*	31 (50.8%)
Subretinal fluid at baseline, *n*	47 (77.1%)
SHRM at baseline, *n*	41 (67.2%)

Quantitative values are expressed in mean ± SD (standard deviation).

BCVA, best-corrected visual acuity; CFT, central foveal thickness; logMAR, logarithm of the minimum angle of resolution; MNV, macular neovascularization; *N*, number; nAMD, neovascular age-related macular degeneration; SHRM, subretinal hyper-reflective material.

The mean BCVA at baseline was 0.5 ± 0.6 LogMAR at baseline and 0.3 ± 0.7 LogMAR at the 12-month follow-up visit (*P* < 0.001). Compared with baseline values, a significant improvement in BCVA was also observed at the 24-month follow-up visit (0.3 ± 0.5 LogMAR, *P* = 0.036). However, no significant differences were found between the 12-month follow-up and the 24-month follow-up visits (*P* = 0.136).

Mean ± SD number of injections in our study cohort was 11.34 ± 1.92 during the 2-year follow-up. Mean ± SD CFT was 333.1 ± 109.8 µm at baseline, 254.6 ± 61.8 µm at the 12-month follow-up visit (*P* < 0.001 versus baseline), and 236.7 ± 57.2 µm at the 24-month follow-up visit (*P* < 0.001 versus baseline and *P* = 0.009 vs. 12-month follow-up visit).

### Qualitative OCT Analysis

At the baseline visit, SRF was observed in 47 eyes (77.1%), whereas the IRF and SHRM were present in 31 eyes (50.8%) and 41 eyes (67.2%), respectively. At the 12-month follow-up visit, SRF was detected in 18 eyes (31.1%), IRF in 9 eyes (14.7%), and SHRM in 7 eyes (11.4%). At the 24-month follow-up visit, SRF was identified in 17 eyes (27.8%), IRF in 5 eyes (8.2%), and SHRM in 5 eyes (8.2%). Regions of cRORA were graded to be present in 31 eyes (50.8%) and 37 eyes (60.6%) at the 12-month and 24-month follow-up visits, respectively.

### HRF Modifications Over Time

**Figure 3.**
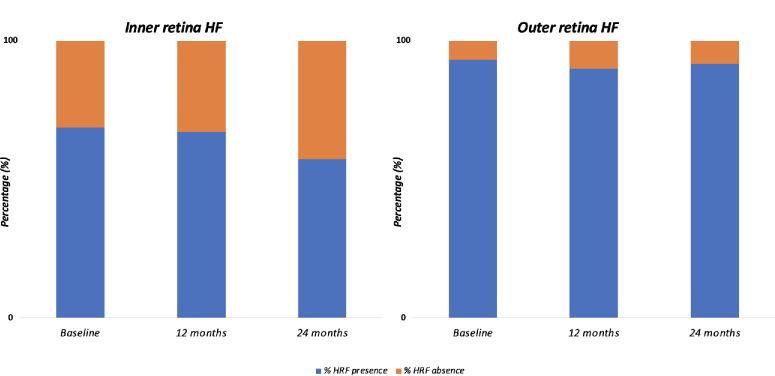
The distribution and evolution of HRFs were assessed at each time point and across different retinal layers.

The percentages of patients exhibiting evidence of HRF at the baseline visit were 68.8% and 93.4% in the inner and outer retina, respectively. The prevalence of HRF was 67.2% and 90.1% at the 12-month follow-up visit, and 57.3% and 91.8% at the 24-month follow-up visit. Figure 3 depicts the modifications in the HRF prevalence throughout the follow-up.

**Figure 4.**
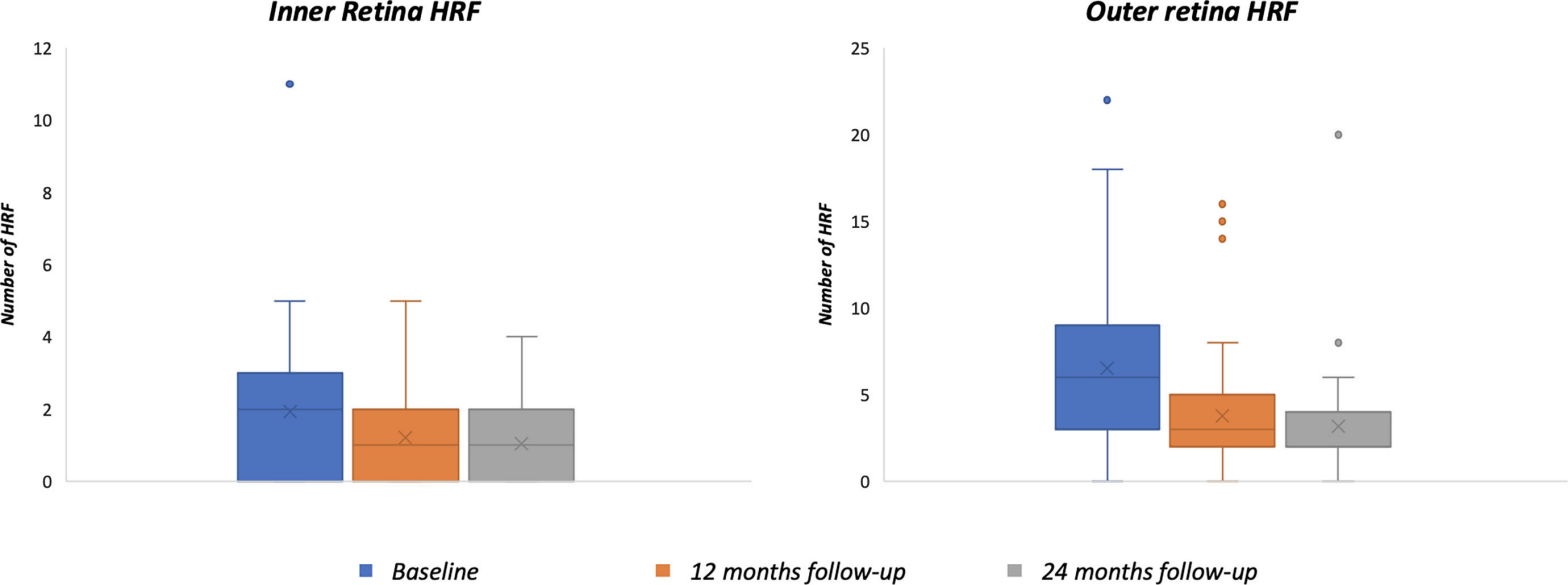
Box-and-whisker plots showing the reduction of HRFs over the follow-up period. At the 12-month follow-up visit, the number of HRFs was significantly reduced at both the inner and outer retina levels compared with baseline values (*P* = 0.002 and *P* < 0.0001, respectively). A similar significant reduction was observed at the 24-month follow-up visit compared to baseline (*P* = 0.002 for the inner retina values and *P* < 0.0001 for the outer retina values). No significant changes were found between the 12-month and 24-month follow-up visits.

The number of HRFs exhibited a significant reduction in amount throughout the follow-up. In details, the number of HRFs was significantly reduced at the 12-month follow-up visit at both the inner and outer retina levels, as compared with baseline values (*P* = 0.002 and *P* < 0.0001, respectively; Fig. 4). Similarly, a significant reduction was found at the 24-month follow-up visit as compared with the baseline visit (*P* = 0.002 for the inner retina values and *P* < 0.0001 for the inner retina values). No significant changes were found between the 12-month and 24-month follow-up visits (Table 2).

**Table 2.** Structural OCT Variables and BCVA Changes Tested at Each Time Visit

	Baseline	12 Mo Follow Up	24 Mo Follow Up
BCVA, logMAR	0.5 ± 0.6	0.3 ± 0.7	0.3 ± 0.5
		*P* < 0.001^a^	*P* = 0.036^a^
			*P* = 0.136^b^
CFT, µm	333.1 ± 109.8	254.6 ± 61.8	236.7 ± 57.2
		*P* < 0.001^a^	*P* < 0.001^a^
			*P* = 0.009^b^
Inner retina HF, *n*	2.0 ± 1.9	1.2 ± 1.1	1.0 ± 1.1
		*P* = 0.002^a^	*P* = 0.002^a^
			*P* = 0.462^b^
Outer retina HF, *n*	6.5 ± 4.1	3.8 ± 3.2	3.1 ± 2.9
		*P* < 0.001^a^	*P* < 0.001^a^
			*P* = 0.180^b^

Data are presented as mean ± standard deviation (SD).

BCVA, best-corrected visual acuity; CMT, central macular thickness; HF, hyper-reflective foci; logMAR, logarithm of the minimum angle of resolution; n, number; SRF, subretinal fluid.

Paired test ^a^ comparison with baseline; ^b^ comparison with 12 months.

### Topographical Correlations for Intraretinal HRF

**Figure 5.**
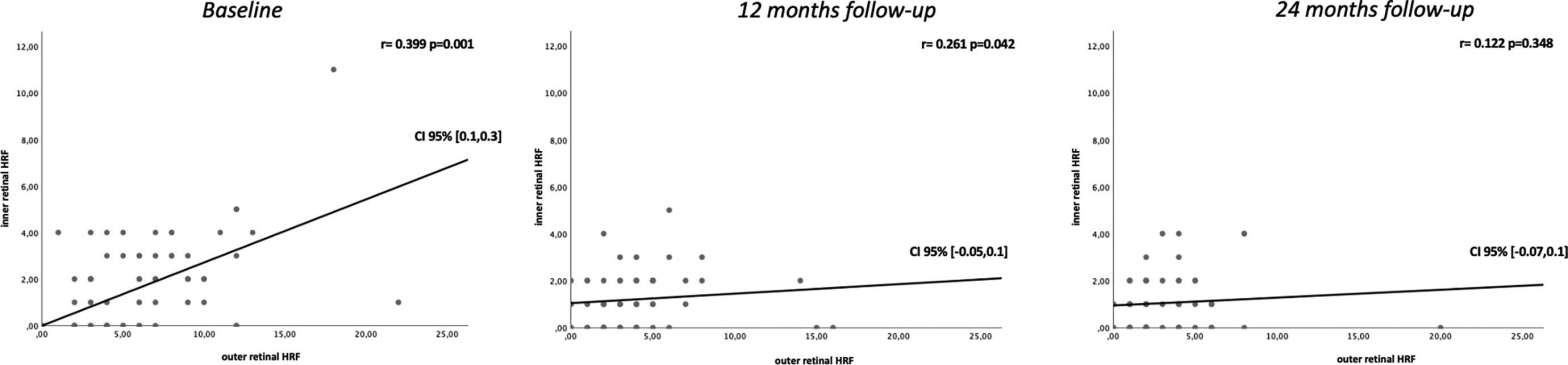
Scatterplots were used to illustrate the correlation between inner and outer retinal HRF at each time point. A significance level of *P* < 0.05 was considered statistically significant. The correlation coefficient was obtained from Pearson rank correlation analysis.

The associations between the topographical amount of HRF in the inner and outer retinal layers were analyzed using Pearson correlation. At the baseline assessment, a notable correlation was observed between HRFs in the inner retina and those in the outer retina (*r* = 0.399, *P* = 0.001). Similarly, at the 12-month follow-up visit, a significant correlation between HRFs in the inner retina and those in the outer retina was found (*r* = 0.261, *P* = 0.042). Conversely, at the 24-month follow-up visit, no HRF correlations between the inner and outer retinal layers were observed (Fig. 5).

### Baseline Risk Factors for Development of Macular Atrophy

Tables 3 and 4 provide a summary of the results from the multivariate regression analysis conducted on the baseline OCT features, along with demographic and clinical characteristics. The most notable association identified regarding the development of cRORA at the 12-month follow-up visit was with the number of HRFs in the outer retina (*P* = 0.039) at the baseline visit. Similarly, a significant association was noted between the development of cRORA at the 24-month follow-up visit and the number of HRFs in the outer retina (*P* = 0.007) at baseline. Subgroup analysis of patients treated with ranibizumab (*n* = 19) and aflibercept (*n* = 42) showed consistent trends in the association between outer retinal HRFs and the development of macular atrophy. In the aflibercept group, the association remained statistically significant (*P* = 0.028 at 12 months and *P* = 0.031 at 24 months). In the ranibizumab group, although the trend was similar, it did not reach statistical significance (*P* = 0.092 at 12 months and *P* = 0.088 at 24 months), likely due to the smaller sample size.

**Table 3.** Results of Multiple Regression Analysis of the Association Between cRORA Development at 12 Months Follow-Up and Other Variables at Baseline

	cRORA as Dependent Variable	
	Standardized ß Coefficient (SE)	*P* Value
Age, y	−0.058 (−0.444)	0.659
Intraretinal fluid at baseline	0.08 (0.611)	0.544
Subretinal fluid at baseline	0.039 (0.298)	0.767
SHRM at baseline	−0.136 (−1.047)	0.299
Number of intravitreal treatments (anti-VEGF), *n*	0.243 (0.029)	0.121
CFT at baseline	0.057 (0.433)	0.667
Outer retinal HF at baseline	0.236 (0.014)	0.039^*^
Inner retinal HF at baseline	0.041 (0.309)	0.758
BCVA (logMAR) at baseline	−0.188 (−1.462)	0.153

Quantitative values are expressed in mean ± standard deviation (SD).

BCVA, best-corrected visual acuity; CFT, central foveal thickness; cRORA, complete RPE and outer retinal atrophy; HF, hyper-reflective foci; logMAR, logarithm of the minimum angle of resolution; n, number; SHRM, subretinal hyperreflective material; SRF, subretinal fluid.

* Statistically significant *P* values are marked with an asterisk sign.

**Table 4.** Results of Multiple Regression Analysis of the Association Between cRORA Development at 24 Months Follow Up and Other Variables at Baseline

	cRORA as Dependent Variable	
	Standardized ß Coefficient (SE)	P Value
Age, y	0.01 (0.075)	0.941
Intraretinal fluid at baseline	0.103 (0.790)	0.433
Subretinal fluid at baseline	0.067 (0.518)	0.612
SHRM at baseline	−0.047 (−0.356)	0.723
Number of intravitreal treatments (anti-VEGF), n	0.318 (0.279)	0.207
CFT at baseline	0.175 (1.355)	0.181
Outer retinal HF at baseline	0.314 (0.014)	0.007^*^
Inner retinal HF at baseline	0.177 (1.373)	0.175
BCVA (logMAR) at baseline	−0.195 (−1.514)	0.136

Quantitative values are expressed in mean ± standard deviation (SD).

BCVA, best-corrected visual acuity; CFT, central foveal thickness; cRORA, complete RPE and outer retinal atrophy; HF, hyper-reflective foci; logMAR, logarithm of the minimum angle of resolution; n, number; SHRM, subretinal hyperreflective material; SRF, subretinal fluid.

* Statistically significative *P* values are marked with an asterisk sign.

### Repeatability

The unweighted k values for intergrader repeatability were 0.96 (59/61) for IRF, 0.98 (60/61) for SRF, and 0.86 (53/61) for SHRM. Agreement was reached for all discrepancies after adjudication between graders.

## Discussion

In this retrospective study, the topographical quantification of HRFs in different retinal layers was conducted and the changes over a 2-year period after the initiation of anti-VEGF treatment in patients with treatment-naïve nAMD at baseline were evaluated. More importantly, the potential role of intraretinal HRFs as a biomarker of development of macular atrophy over time was investigated. Overall, after 1 year of anti-VEGF therapy, a noteworthy decrease in HRF amount was found and this trend persisted even after 2 years of anti-VEGF therapy. Furthermore, the quantity of HRFs in the outer retina emerged as the most robust predictor for the development of macular atrophy at both 12-month and 24-month follow-up visits.

Intraretinal HRFs have been detected on structural OCT images and are associated with several conditions including retinal venous occlusion,^29^ retinitis pigmentosa,^30^ central serous chorioretinopathy,^31^ and diabetic macular edema.^32^^,^^33^ Importantly, HRFs seem to play a predominant role in AMD even during the early stages of the disease. In these eyes (i.e. early and intermediate AMD), HRFs could potentially represent migrated RPE cells, microglia, or extravasated materials. In the study from Christenbury et al.^34^ using structural OCT, HRFs were identified in eyes with intermediate AMD and were demonstrated to increase in number over time. Moreover, the distribution of HRFs gradually but significantly shifted from the RPE and outer retina to the inner retinal layers during the follow-up period, this suggesting longitudinal changes occurring to these OCT findings. Nevertheless, it is crucial to emphasize that HRFs exist in various retinal layers, and they may originate from different sources. Therefore, the topographical quantification of HRFs in AMD eyes based on their location could hold clinical significance.

In agreement with Hsia and colleagues,^27^ our current study also indicates that the outer retina harbors the largest number of HRFs. Additionally, aligning with the same findings, we observed a notable decrease in HRFs across all layers following anti-VEGF therapy. It is important to highlight that our follow-up duration spans 2 years, revealing that the most substantial reduction tends to occur after the first year of treatment.

Inspired by the above-mentioned findings, we sought to provide a topographical analysis of the HRFs in the inner and outer retina. Therefore, we examined the correlation of HRF in each layer with the development of macular atrophy at 12 and 24 months. This exploration aimed at identifying prognostic factors for the development of cRORA in nAMD. We found that the most influential and reliable predictor or precursor lesion for the development of macular atrophy following anti-VEGF therapy was the amount of HRFs in the outer retina. These results align with earlier studies showing that the amount of intraretinal HRFs is associated with a poor visual prognosis in nAMD eyes with type 1 MNV.^17^

Nevertheless, most studies have focused on examining the correlation between HRF and the progression to macular atrophy, particularly beginning from the intermediate stages of AMD. Leuschen et al.^35^ have suggested that the correlation or colocalization of HRF with the drusen apex in intermediate AMD demonstrates an increased association with RPE atrophy at baseline. Additionally, when macular atrophy is not initially present, this association is linked to a heightened risk of atrophy developing at that location. In a recent study, Au et al.^36^ illustrated that eyes with taller drusen or drusenoid PED exhibited a propensity to display other elevated features associated with the progression to atrophy, including intraretinal HRF.

Hence, this study signifies a noteworthy enhancement in the existing literature, being the first to assert that the quantity of HRFs in the outer retina at baseline acts as an indicator for the development of macular atrophy following anti-VEGF treatment. These findings underscore the significance of not only the presence but also the HRF topographical distribution in terms of the risk for progression to cRORA. Macular atrophy is a common complication that may be observed in eyes with nAMD and is typically associated with worse visual outcomes.^37^ Previous studies have consistently demonstrated that the presence of HRFs in the outer retina is associated with disrupted outer segments and a subsequent decline in visual acuity.^38^^,^^21^ Moreover, there is a suggestion that HRFs in the outer retinal layers may be more likely to represent distressed RPE cells, which are recognized as one of the various phenotypes of distressed RPE cells, as described by Curcio and colleagues.^39^ Therefore, it is perhaps unsurprising that cRORA frequently occurs in this particular location. Dysfunction of the RPE is recognized to lead to atrophy of the underlying choriocapillaris.^40^ In the presence of choriocapillaris atrophy, upregulation of vascular endothelial growth factor secretion occurs, which, in turn, induces reactive proliferation and migration of RPE cells.^41^ This observation may imply that outer retina HRFs represent a subsequent phenotype, precursor, or characteristic in AMD associated with type 1 MNV. This manifestation appears to be more proximal to the time of atrophy development following anti-VEGF therapy. The evolving correlation between inner and outer retinal HRFs over time provides interesting insights into the dynamic nature of these lesions. The initial significant correlation at baseline suggests a diffuse process affecting multiple retinal layers in treatment-naïve nAMD. However, the weakening of this correlation at 12 months and its loss of significance at 24 months might indicate a differentiation in the fate of HRFs in different retinal layers following anti-VEGF treatment.

Importantly, in the spectral-domain OCT (SD-OCT) grading process for the current study, we incorporated additional HRFs present in various retinal layers that, in our assessment, did not align with transformed or migrating RPE cells on SD-OCT. In nAMD, MNV gives rise to subretinal fluid and fibrovascular membranes, potentially resulting in numerous punctate hyperreflective specks within the intraretinal and subretinal space. The MNV process involves the formation of abnormal, fragile blood vessels that leak into subretinal and intraretinal spaces, and the hyper-reflectivities are likely indicative of lipid exudates or inflammatory crystalline deposits arising from vascular leakage.

The present study has a main limitation related to its retrospective design, preventing the assessment of HRF repeatability. Additionally, only horizontal OCT scans centered on the fovea were examined. Other constraints include the manual counting of HRFs in each layer, which is a time-consuming process and impractical for routine clinical use. Moreover, our study exclusively considered a predefined set of features for evaluation during the baseline visit. Another limitation of our study is that we did not conduct a detailed topographical analysis of the co-localization between baseline HRF and subsequent cRORA development. Our study did not analyze the relationship between cRORA and outer retinal features, such as ELM and ellipsoid zone (EZ) integrity, as this was beyond our initial protocol. Furthermore, the use of a single OCT scan through the fovea would not have provided sufficient data for a comprehensive co-localization analysis across the entire macula. Finally, whereas our subgroup analysis of ranibizumab and aflibercept-treated patients showed consistent trends, the smaller sample size in the ranibizumab group led to some loss of statistical power. Moreover, the requirement for a minimum number of anti-VEGF injections may have introduced selection bias. Despite these limitations, we utilized an established classification system to categorize the number of HRFs into distinct levels. This strategy streamlines interpretation, offering a faster assessment and diminishing reliance on individual observers. We acknowledge that our reliance on manual HRF counting by retinal specialists introduces the possibility of interobserver variability. Although we reported high intraclass correlation coefficient, future studies could benefit from the implementation of artificial intelligence algorithms to enhance the efficiency and precision of HRF counting. This advancement may enable the analysis of various OCT sections beyond foveal-centered cross-sections.

In conclusion, HRFs in different retinal layers demonstrated diverse clinical courses in patients with nAMD, suggesting a potentially heterogeneous origin. Following just 1 year of antiangiogenic therapy, a notable reduction in HRFs was observed in the inner and outer retinal layers, persisting even after 2 years of anti-VEGF treatment. Importantly, the quantity of HRF in the outer retina at baseline was found to be correlated with the development of macular atrophy, maintaining significance at both 1 and 2 years after anti-VEGF treatment. This suggests that the presence of HRFs in the outer retinal layer at baseline, along with its changes post-treatment, may serve as a potential biomarker in patients with nAMD.
